# 

**Published:** 1876-02

**Authors:** 


### Table of Contents.

### ORIGINAL COMMUNICATIONS.

A CASE OF SKIN GRAFTING. *By B. M. Cromwell, M.D.* ........ 641
MAMMITIS AND MAMMARY ABSCESSES TREATED BY BANDAGING. *By L. A. Dugas, M.D., LL.D.* ........ 646
MURIATED TINCTURE OF IRON IN FLUOR ALBUS. *By J. M. Johnson, M.D.* 650
OPIUM POISONING. *By J. M. Johnson, M.D.* ........ 653

### ATLANTA ACADEMY OF MEDICINE.

Failure of Lochial Discharge after delivery—Lobulated Tumor—Strangulated Inguinal Hernia—Acute Urethritis—Hemi-Chorea—Extirpation of the Eye-ball—Opium Poisoning—Impaction of the Cecum—Double Uterus—Veratrum an Antidote to Opium ........ 655

### CLINIC ATLANTA MEDICAL COLLEGE.

Eye and Ear, A. W. Calhoun, M.D ........ 670
Services of Prof. W. F. Westmoreland, M.D ........ 673

### OUR EXCHANGES.

Clinical conversations with his Private Classes at Demlit Dispensary, by L. Duncan Bulkley ........ 679
Total Destruction of one Lung ........ 687
Excision of the Uvula and Tonsils ........ 688
Treatment of the Vomiting of Pregnancy by Mechanical Appliances.... 688
Salicylic Acid in Otorrhœa ........ 689
Bacteria and Septicæmia ........ 689
An Agreeable Cathartic ........ 690
Milk Kept by Chloroform ........ 690
Rheumatic Fever and its Treatment ........ 691
Syphilitic Infection by the Semen ........ 691
Retained Placenta ........ 692
Vaginismus ........ 692
Abortion in Japan ........ 693
Rare case of Vaccination ........ 693
How long may the Efficacy of Vaccination be relied on ........ 693
A Horrible Labor ........ 694
A Case of Triplets ........ 694
Silicate of Potash ........ 695
Gleanings ........ 695

### BIBLIOGRAPHICAL.

Medical Diagnosis with Special Reference to Practical Medicine ........ 698
The Body and its Ailiments ........ 699
Pamphlets ........

### EDITORIAL.

Georgia State Board of Health ........ 700
The Jealousies and Bickerings in the Profession ........ 702

